# Magnitude of erectile dysfunction and associated factors among adult diabetic men on follow-up at Goba and Robe hospitals, Bale Zone, South East Ethiopia: hospital-based cross-sectional study

**DOI:** 10.1186/s12902-023-01489-x

**Published:** 2023-10-26

**Authors:** Telila Mesfin, Yohannes Tekalegn, Ahmednur Adem, Kenbon Seyoum, Girma Geta, Biniyam Sahiledengle, Eshetu Mesfin, Demisu Zenbaba, Fikreab Desta, Girma Beressa, Mesfin Tsegaye, Neway Ejigu, Degefa Gomora

**Affiliations:** 1 Department of Medicine, School of Health Sciences, Madda Walabu University, Goba, Ethiopia; 2 Department of Public Health, School of Health Sciences, Madda Walabu University, Goba, Ethiopia; 3 Department of Midwifery, School of Health Sciences, Madda Walabu University, Goba, Ethiopia; 4 Department of Public Health, ICAP, Addis Ababa, Ethiopia

**Keywords:** Prevalence, Erectile dysfunction, Diabetic, Ethiopia

## Abstract

**Purpose:**

Erectile dysfunction is defined as the inability to achieve and/or maintain an erection of sufficient rigidity and duration to permit satisfactory sexual performance. The purpose of this study is to assess the prevalence of erectile dysfunction and associated factors among adult diabetic men on follow-up at Goba and Robe hospitals, Bale Zone, South East Ethiopia,2022.

**Methods:**

Hospital-based cross-sectional study design was used among 420 adult diabetic men from March 1 to April 30 using a systematic random sampling technique. An international index of erectile function questionnaire containing five questions was used to assess the outcome variable. The data were entered, edited, and coded using Epidata version 4.6 and analyzed using SPSS version 26. Bivariable and multivariable binary logistic regression analysis were performed to identify factors associated with erectile dysfunction. Adjusted odds ratios with their corresponding 95% confidence interval were computed to estimate the strength of association. Statistical significance was declared at p-value < 0.05.

**Results:**

The prevalence of erectile dysfunction was found to be 354 (84.3%). Multivariable logistic regression revealed that erectile dysfunction is significantly associated with old age (AOR = 12.39, 95% CI:5.10–30.08), inadequate physical activity (AOR = 4.15, 95% CI:1.33–12.97), and being rich (AOR = 2.62, 95% CI = 1.21–5.66).

**Conclusion:**

The prevalence of erectile dysfunction in this study population is nearly nine out of ten. Age, inadequate physical activity, and wealth index were independent predictors of erectile dysfunction. Assessment and management of erectile dysfunction in diabetic clinics should be routine medical care.

**Supplementary Information:**

The online version contains supplementary material available at 10.1186/s12902-023-01489-x.

## Introduction

Erectile dysfunction(ED) is defined as the inability to achieve and/or maintain an erection of sufficient rigidity and duration to permit satisfactory sexual performance [1]. The degree of dysfunction has been used to classify erectile performance, and estimates of prevalence (the number of men who have the problem) vary depending on the definition of erectile dysfunction utilized [2]. Diabetes mellitus causes a number of short-term and long-term complications [3–8]. From long-term complications, erectile dysfunction is one of them [9–11]. Endothelial dysfunction, accumulation of advanced glycation end products, oxidative stress, and autonomic neuropathy are all linked to the pathogenesis of erectile dysfunction in diabetic patients. For example, diabetes may alter cavernous nerve impulses and endothelial cells, resulting in a neurotransmitter deficit [12, 13].

Erectile dysfunction has a major impact on job productivity and quality of life. Better management and early detection could help alleviate this cost, particularly in nations where erectile dysfunction is linked to poor economic and health consequences [14]. The 2018 American Urological Association clinical guidelines for erectile dysfunction recommend a shared decision-making approach between the physician and patient for the various treatment options. Erectile dysfunction remains an underdiagnosed and undertreated condition associated with many healthcare conditions [15–18]. Providers are missing a significant chance to improve their patients’ quality of life by failing to recognize erectile dysfunction. Even though, erectile dysfunction has a detrimental impact on diabetes patients’ lives, frank discussion of sexually related issues is frowned upon in Ethiopia. As a result, sexual disorders in Ethiopia are under-recognized and under-treated [19].

Sexual function is a key indicator of life satisfaction. Men’s social interactions, emotional and psychological well-being, and partner relationships have all been shown to be negatively impacted by erectile dysfunction [20]. According to the literature, about 95% of cases of erectile dysfunction can be successfully treated [21]. Erectile dysfunction is associated with mortality, but the link between low testosterone and greater mortality is still debatable. Low testosterone and sexual dysfunction frequently coexist, although it is unknown how important low testosterone is in predicting death compared to sexual symptoms [22]. Over 50% of men between the ages of 40 and 70 experience erectile dysfunction, which is a common health issue in older men [23]. Even though more common as people get older, it is not a natural aspect of the aging process. Older men are more likely to develop testosterone deficiency, especially those who are obese, have type 2 diabetes, chronic kidney disease (CKD), and other comorbid conditions, such as acute COVID-19 infection and its long-term effects, as well as those who are taking medications, particularly opiates, anabolic steroids, antipsychotics, and anticonvulsants [24]. Erectile dysfunction isn’t just about being unable to have satisfying sexual intercourse, it is frequently associated with emotional and psychological stigma [1, 25].

A couple of years ago, erectile dysfunction was one of the most neglected complications of diabetes [25]. In the past, physicians and patients were led to believe that declining sexual function was an inevitable consequence of advancing age or was brought on by emotional problems. This myth, combined with men’s natural reluctance to discuss their sexual problems and physicians’ inexperience with sexual issues, failed to directly address this problem with the majority of patients experiencing it [26]. Erectile dysfunction tends to occur earlier in diabetic patients than in normal individuals [7, 27]. According to several kinds of research, erectile dysfunction has both psychological and social impacts on men, including a loss of confidence in their lifestyle, anxiety, depression, loss of personal relationships, significant repercussions on their self-esteem, and a loss of social and work activities [28, 29]. The problem is a huge burden behind divorce, especially for young couples [30].

A variety of studies have been conducted to determine the magnitude of erectile dysfunction and its determinants [26, 31–36]. However, the wide scope of the problem necessitates studying and analyzing the various factors that are potentially associated with erectile dysfunction at once [37, 38]. In addition to the variables covered in earlier studies, wealth-related variables are considered in this study. In the past, researchers have tried to investigate the problem [26, 28, 36, 38–41]. In particular, no research has been conducted in Southeastern Ethiopia. Therefore, this study aims to determine the magnitude of erectile dysfunction and associated factors among adult diabetic men on follow-up at Goba and Robe hospitals.

## Materials and methods

### Study area and period

The study was conducted at Goba and Robe hospitals in Oromia region, Bale zone which is located 435 km to the southeast of Addis Ababa, Ethiopia. Goba Referral Hospital is the only referral hospital in the Bale zone that serves as a teaching hospital for medical and health sciences. Data was collected from March 1 to April 30, 2022 by two trained male nurses.

### Study design

Hospital-based cross-sectional study design.

### Source population

All diabetic men who visit chronic outpatient departments of Goba and Robe Hospitals.

### Study population

All adult diabetic men who fulfill eligibility criteria and have follow-up at Goba and Robe hospitals during the data collection period.

### Eligibility criteria

#### Inclusion criteria

Adult diabetic men aged ≥ 18 years who also had a history of sexual intercourse.

#### Exclusion criteria

Critical patients with hyperosmotic hyperosmolar state or diabetic ketoacidosis, mentally incompetent, Psychotic patients, Patients with previous pelvic surgery, Patients who had pelvic trauma, Patients who had a spinal injury, Patients taking anti-psychotic medications, patients with any malignancies and patients with benign prostatic hyperplasia were excluded from the study through history taking and physical evaluation.

### Sample size determination

The study sample size was determined using epi-info 7 based on assumptions of 95% confidence level, 5% margin of error, the proportion of erectile dysfunction 54.3% [19], and non-response rate of 10%. The maximum sample size was obtained for 54.3% proportion. Taking a non-response rate of 10%, the final sample size is 420.

### Sampling technique

A systematic random sampling technique was used to select study subjects. The average number of monthly adult diabetic cases seen in the outpatient department of Goba and Robe hospitals within the preceding six months were taken (from Goba 600 and Robe 460) and divided by proportionally allocated sample size (238 for Goba and 182 for Robe hospitals). When we divide this by the allocated sample size for each hospital the quotient is the interval chosen to select each case. Therefore, the respondents were interviewed every 2 and 3 patients for Goba and Robe hospitals respectively. The medical record number was taken for each patient to avoid duplication.

### Variables

#### Dependent variable

Erectile dysfunction is the outcome variable for this study. It was assessed using International Index of Erectile Function (IIEF**)** questionnaire. This questionnaire consists of five questions, each with Likert scale alternative responses. The greatest possible score is 25, which is the score for people who never had an erection problem and the lowest score is 5. The sum of the ordinal replies to the 5 items determines the IIEF-5 score. The erectile dysfunction was categorized into two depending on the IIEF score. Those who score 22 and above were classified as having no erectile dysfunction and those who score 21 and below were classified as having erectile dysfunction. More specifically, those who have a score of 17–21, 12–16, 8–11, and 5–7 are sub-categorized as having mild, mild to moderate, moderate and severe erectile dysfunction respectively [42].

#### Independent variables

##### Socio-demographic and economic characteristics

Age, Place of residence, Marital status, Ethnicity, Religion, Occupation, Educational status, Income.

##### Wealth index

Principal component analysis was done for common household assets. The requirements for the analysis like large sample size, the ratio of cases to variables, KMO > 0.6, and Bartlett test of sphericity statistical significance(P < 0.05) were checked and satisfied. Accordingly, KMO = 0.601 and Bartlett’s test of sphericity < 0.001. Four variables number of chicken, radio, refrigerator, and wrist watch with eigen value greater than one were extracted and explained 24.29%, 18.17%, 13.5%, and 12.77% respectively. The total wealth index variability explained by principal component analysis was 68.74%.

##### Treatment and lifestyle

Types of diabetes, Duration of diabetes, Types of treatment, BMI, Waist circumference, Hip circumference, Waist to hip ratio, Smoking, Alcohol, Khat chewing, Physical activity, Diet.

##### Comorbidities and Complications

Hypertension, Antihypertensive medications, Cardiac disease, Cardiac drugs, Eye disease, Renal disease, Stroke, Peripheral neuropathy.

##### Mental and psychological factors

Depression.

### Operational definition

#### PHQ-2 and PHQ-9(patient health questionnaire)

were used to assess variable depression. The PHQ-2 inquiries about the frequency of depressed mood and anhedonia over the past two weeks. It includes the first two items of the PHQ-9. The purpose of the PHQ-2 is to screen for depression in a “first-step” approach. Patients who screen positive or have a score of ≥ 3 should be further evaluated with the PHQ-9 to determine whether they meet the criteria for a depressive disorder [43]. PHQ-2 score ranges from 0 to 6 [44]. PHQ-9 tool contains nine questions with four options of answers. Each score was summed up and labeled accordingly [45]. For this study, score of 10 and above were used to define depression with good sensitivity.

#### International Physical Activity Questionnaire (IPAQ)

This assesses the types of the intensity of physical activity and sitting time that people do as part of their daily lives are considered to estimate total physical activity in MET-min/week and time spent sitting [46]. The total number of days and time in minutes spent on activities within a week will be multiplied by their corresponding MET vigorous, moderate, and walking activities. The outcome was categorized and named as high (At least 3000 MET), moderate (At least 600 MET but less than 3000 MET) and low (unable to classify as high or moderate) for vigorous, moderate, and walking activities respectively.

#### Waist circumference

Is a measurement of abdominal obesity and provides independent risk information that is not accounted for by body mass index. It is measured with flexible tape placed on a horizontal plane between anterior superior iliac spine and the lower rib. A normal waist circumference for male should be less than 102 cm [47].

#### Waist-to-hip-ratio (WHR)

Measures the ratio of waist circumference to hip circumference. It determines how much fat is stored on waist, hips, and buttocks. Even though, the cut off value vary from population to population a WHR of 1 and above increases risk of metabolic syndrome [48].

### Data collection tool and procedure

After reviewing different works of literature [41, 42, 49–51], a written structured questionnaires were prepared to determine the prevalence of erectile dysfunction and associated factors among adult diabetic men at Goba and Robe hospitals. The English version of the questionnaire was translated into Afan Oromo and Amharic, the local languages spoken in the study area. After the translation, the questionnaire was -translated back to English to check the consistency. Data were collected through interviewer administered questionnaire and physical measurements. The standard tools used for this study were WHO STEPS-wise questionnaires, summary of diabetes self-care activities, international physical activity questionnaire, public health questionnaires, international index of erectile function and Ethiopian demographic and housing survey data. The selection of these hospitals was made based on patient flows and the comprehensive service they provide. The data were collected by interviewing the patients individually by two trained male nurses on working days. The international index of erectile function questionnaires was used to screen the patients for erectile dysfunction. A separate and quiet room was used for data collection. The remaining data were retrieved from the patients’ documents using prepared checklists.

### Data quality control

The principal investigator gave training for two male nurses before the data collection period. The data collectors were capable of speaking local languages. The training was given on the objective of the study, actual data collection procedure, the contents of the questionnaires, and matters related to confidentiality. Additionally, to maintain the quality of data pretest was conducted by the principal investigator at Ginnir hospital on 5% of the sample size to check the validity and know whether the questionnaires are understandable or not a week before beginning the actual data collection period. The modification was made to the questionnaire that is found with any ambiguity and affects its consistency. During data collection, the principal investigator gave onsite technical support and close supervision. Data were checked daily by the principal investigator for completeness, and consistency and then errors were corrected accordingly before data processing and analysis.

### Data processing and analysis

Data were edited, coded and entered by Epidata version 4.6 and were exported to SPSS (version 26) for analysis. The data analysis started from a basic description to the identification of potential factors associated with erectile dysfunction. Bivariable and multivariable logistic regression were used to identify the relationship between erectile dysfunction and various factors. The assumptions of the logistic regression model Hosmer-Lemeshow goodness of fit statistics was checked and satisfied. Multivariable logistic regression was used to identify potential confounding variables. Multicollinearity among independent variables were checked using tolerance and variance inflation factor. Normality for independent variables was checked using Shapiro wilk. Principal component analysis was performed for common household assets. Descriptive summaries were computed as simple frequencies, mean, median and standard deviations. All explanatory variables which resulted in p < 0.25 with the outcome variable in the bivariable were entered into a multivariable logistic regression model to identify factors associated with erectile dysfunction. P-Value < 0.05 was considered statistically significant and the adjusted odds ratio with a 95% confidence interval was used to declare association.

## Results

### Socio-demographic characteristics

A total of 420 adult diabetic men were interviewed successfully with a 100% response rate. The median age of the study participants was 52 years ranging from 18 to 85 years. Three hundred and thirty-one (78.8%) of the respondents were currently married and three hundred and forty-nine (83.1%) of the respondents were from the Oromo ethnic group. Half of the study participants were Muslim 215 (51.2%) followed by Orthodox Christians. The occupations of the respondents were mainly farmers 125 (29.8%) and government employees 74 (18.6%). The educational status of the respondents was primary (5–8), secondary (9–10), higher (bachelor’s degree and above) with a corresponding percentage of 82 (19.5%), 75 (17.9%), and 67 (16%) respectively. The median monthly income of the study participants was 1000**(**Table 1**)**.

**Table 1:** Socio-demographic characteristics of study participants at Robe Hospital and Goba Referral Hospital, 2022 (n = 420)

Variable		Frequency	Percent
**Age in years**	< 30	78	18.6
	30–44	78	18.6
	45–59	112	26.6
	≥ 60	152	36.2
**Place of residence**	Urban	274	65.2
	rural	146	34.8
**Marital status**	Never married	51	12.1
	Currently married	331	78.8
	divorced	13	3.1
	widowed	12	2.9
	cohabitating	13	3.1
**Ethnicity**	Oromo	349	83.1
	Amhara	71	16.9
**Religion**	Orthodox Christian	199	47.4
	Protestant	5	1.2
	Muslim	215	51.2
	Catholic	1	0.2
**Occupation**	Merchant	38	9
	Farmer	125	29.8
	Government employee	74	17.6
	Private or NGO	12	2.9
	Self-employed	66	15.7
	Student	24	5.7
	Daily laborer	12	2.9
	Retired	43	10.2
	Unemployed	26	6.2
**Educational status**	Don’t read and write	20	4.8
	Only read and write	48	11.4
	Primary (1–4)	41	9.8
	Primary (5–8)	82	19.4
	Secondary (9–10)	75	17.9
	Preparatory (11–12)	66	15.7
	Diploma (technical or vocational)	21	5
	Higher (bachelor or above)	67	16
**Monthly income**	< 500 birr	133	31.7
	≥ 500 birr	287	68.3

### Medical condition, BMI, physical activity, and mental status of adult diabetic men attending diabetic clinic at Robe and Goba Hospitals (n = 420),2022

Among respondents more than half of them were diagnosed with type 2 diabetes mellitus 287(68.3%). The median duration of diabetes mellitus was 5 years with interquartile range (IQR) of 2–8 years. Two hundred and eighteen (51.9%) of the study participants use oral glucose lowering agents followed by insulin 173 (41.2%) and combination therapy 29 (6.9%). The mean fasting blood sugar of the respondents is 153.55(SD ± 63.9) and 189 (45%) of them had good glycemic control (FBS ≤ 130). The leading DM related complication was hypertension 143 (34%) followed by cardiac 19 (4.5%), renal 6 (1.4%), eye 6 (1.4%), and peripheral neuropathy 1(0.2%). The average BMI of the respondents was 24.02(SD ± 3.52). Overweight and obese accounts for 27.4% and 6.9% respectively. Three hundred and twenty-nine (78.3%) of the respondents had a waist circumference of < 102 cm and two hundred and sixty-six (63.3%) of them had waist-to-hip-ratio < 1(Table 2).

**Table 2:** The medical condition, BMI, and physical activities of adult diabetic men attending diabetic clinic at Robe and Goba hospitals,2022

Variable		Number	Percent
Diabetes duration	< 5 years	225	53.6
	5–10 years	129	30.7
	> 10 years	66	15.7
Types of diabetes	Type 1	133	31.7
	Type 2	287	68.3
Medication	Insulin	173	41.2
	Oral hypoglycemics	218	51.9
	Combination	29	6.9
Fasting blood sugar	≤ 130 mg/dl	189	45
	> 130 mg/dl	231	55
**Diabetic complications**			
Hypertension	Yes	143	34
	No	277	66
Cardiac	Yes	19	4.5
	Not reported	401	95.5
Renal	Yes	6	1.4
	Not reported	414	98.6
Eye	Yes	6	1.4
	Not reported	414	98.6
Peripheral neuropathy	Yes	1	0.2
	Not reported	419	99.8
**BMI and physical activities**			
BMI	< 18.5 kg/m^2^	12	2.9
	18.5-24.99 kg/m^2^	264	62.9
	25-29.99 kg/m^2^	115	27.4
	≥ 30 kg/m^2^	29	6.9
Waist circumference	< 102 cm	329	78.3
	≥102 cm	91	21.7
Waist-to-hip-ratio	< 1	266	63.3
	≥ 1	154	36.7
Physical activity	Low (less than 600 MET)	143	34
	Moderate (At least 600 but < 3000 MET)	195	46.4
	High (At least 3000 MET)	82	19.5
Depression	Yes**	65	15.5
	No*	355	84.5

Key: * score of PHQ-9 = 0–9, **= score of PHQ-9 = ≥ 10

### Behavioral and lifestyle characteristics of adult diabetic men attending diabetic clinic at Robe and Goba Hospitals(n = 420), 2022

Among 420 respondents only 6 (1.4%) smoke tobacco products currently and 5 (1.2%) of them smoke tobacco products daily. The average age at first started smoking daily is 31.67 (SD ± 8.14) and it happened 35.23 (SD ± 6.28) years ago. Sixty-four (15.2%) out of 420 respondents were past smokers and the median age at stopping cigarettes was 30 years. Regarding alcohol consumption 143 (34%) ever consumed alcoholic drinks. The median number of occasions of having at least one alcoholic drink during the past 30 days is two. On the other hand, the median standard alcohol drinks during the past 30 days are two. The median number of having the largest standard alcoholic drinks counting all types of drinks during the past 30 days is four. Likewise, the median number of having five or more standard alcohol drinks on a single occasion in the past 30 days is zero.

Regarding fruit and vegetables, the median number of days of fruit and vegetable consumption is 1 and 2 respectively. The median number of servings of fruits and vegetables in a typical week is 1. The median number of meals eaten that were not prepared at home per week is zero. More than half of the participants 240(57.1%) don’t skip their breakfast in a typical week and the most commonly used oil for meal preparation was vegetable oil 373 (88.8%).

### Prevalence of erectile dysfunction

**Fig. 1:**
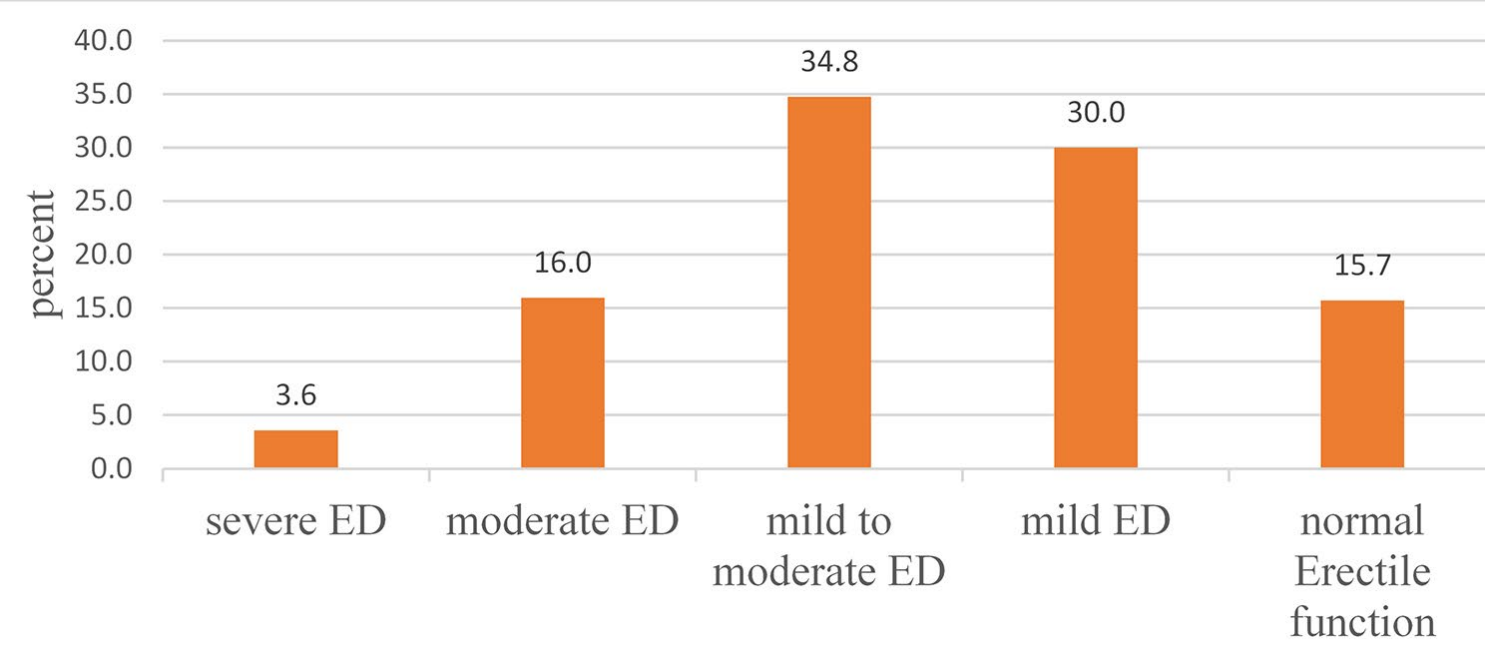
Erectile dysfunction category among adult diabetic men attending diabetic clinic at Robe and Goba Hospitals

The mean score of erectile dysfunctions based on IIEF was 16.17(SD ± 4.65) ranging from 5 to 25. The prevalence of erectile dysfunction was found to be 354 (84.3%) of this 126 (30%) had mild erectile dysfunction,146 (34.8%) had mild to moderate erectile dysfunction, 67 (16%) had moderate erectile dysfunction and 15 (3.6%) had severe erectile dysfunction. The remaining 66 (15.7%) had normal erectile function (Fig. 1).

### Factors associated with erectile dysfunction

Variables like age, physical activities, types of diabetes, presence of hypertension, waist to hip ratio, waist circumference, and wealth index had a p-value of less than 0.25 in bivariable logistic regression and hence exported to multivariable logistic regression analysis for controlling possible confounders. Multivariable binary logistic regression analysis showed age, physical activities, and wealth index to be statistically associated with erectile dysfunction. More specifically, the odds of having erectile dysfunction among those who are ≥ 45 years old are about 12.39 times higher compared to young age groups (AOR = 12.39 ,95% CI = 5.10-30.08, p-value = < 0.001). Likewise, rich diabetic men are 2.62 times at higher risk of having erectile dysfunction compared to poor (AOR = 2.62, 95% CI = 1.21–5.66, p-value = 0.014) (Table 3).

**Table 3:** Factors associated with erectile dysfunction among adult diabetic men attending diabetic clinic at Robe and Goba Hospitals, 2022

		Erectile dysfunction		COR (95% CI)	AOR (95% CI)	P-value
		No	Yes			
Age	less than 30	37	41	1	1	
	30–44	16	75	4.23(2.10–8.51)	3.49(1.65–7.37)	0.001*
	45 and above	13	238	16.52(8.09–33.72)	12.39(5.10-30.08)	**<0.001** ^*^
Wealth index	poor	38	104	1	1	
	medium	15	91	2.21(1.14–4.29)	2.01(0.94–4.28)	0.068
	rich	13	159	4.46(2.27–8.79)	2.62(1.21–5.66)	0.014*
Waist circumference	high	8	83	2.22(1.02–4.83)	0.64(0.24–1.65)	0.358
	normal	58	271	1	1	
Waist-to-Hip Ratio	high	14	140	2.43(1.29–4.55)	1.18(0.51–2.76)	0.693
	normal	52	214	1	1	
Hypertension	no	52	225	1	1	
	yes	14	129	2.13(1.14–3.99)	0.46(0.20–1.05)	0.066
Types of Diabetes	type 1	46	87	1	1	
	type 2	20	267	7.05(3.96–12.58)	1.59(0.61–4.18)	0.343
Physical activity	low	5	138	8.32(2.97–23.29)	4.15(1.33–12.97)	0.014*
	moderate	42	153	1.09(0.59–2.03)	0.9(0.45–1.80)	0.777
	high	19	63	1	1	

**Key: -** *=p-value < 0.05

## Discussion

This study assessed the prevalence and factors associated with erectile dysfunction among adult diabetic men on follow-up at Robe and Goba hospitals. In this particular study, the prevalence of erectile dysfunction among adult diabetic men was 354 (84.3%). This finding is in line with research conducted in the Amhara region of Ethiopia 85.5% [41], in Ghana 81.8% [52] and Indonesia 84.4% [53]. Moreover, this study found a higher prevalence compared to researches done in most areas of Ethiopia(54.3-69.9%) [19, 39, 54, 55], Eritrea 74.2% [56] ,Tanzania 55.1% [50], recent systematic review done in Africa 71.45% [57], Western Asia 62.2% [58], China 64.2% [40], South America 74.6% [59], Bangladesh(45.3-60.2%) [35, 60], Sri Lanka 62.9% [51], Pakistan 21.1% [31], Nepal 76.8% [61], India 68.5% [27] and Europe 67.4% [62]. This might be due to the study participants characteristics like older age and higher fasting blood sugar. Therefore, poor adherence to diabetic medications and older age may be responsible for the observed higher prevalence. Consequently, it becomes essential to undertake appropriate and proactive management of diabetes mellitus such as, healthy lifestyle choices, and regular glycemic monitoring, to minimize the occurrence of adverse health events associated with diabetes, including erectile dysfunction. In addition, the finding raises the issue of diabetic medication potency and storage areas. This can be due to the high fasting blood sugar level in more than half of the study participants. Similarly, insulin is a protein hormone that can be sensitive to temperature changes. Extreme heat or freezing temperatures can degrade the insulin molecules and render them ineffective. Even though most of the patients are from urban areas, they don’t have proper storage areas like refrigerators. On the contrary, the prevalence of erectile dysfunction is a bit lower compared to the studies conducted in Morocco (88%) and Nigeria (94.7%) [63, 64]. The possible reason may be attributed to the socio-cultural context of the study participants, study design, and sample size.

The factors associated with erectile dysfunction in this study are age ≥ 45, inadequate physical activities (< 600 MET), and being rich. This study showed that being older (≥ 45 years) is 12.39 times more likely to develop erectile dysfunction which is in line with most studies [51, 53, 54, 56, 65]. This can be due to age-related physiological changes like decreased testosterone production in older men [66].

In this study, less or inadequate involvement in physical activities showed a strong association with erectile dysfunction. Adult diabetic men with inadequate physical activities are more than threefold likely to develop erectile dysfunction. This finding is supported by studies conducted in Ethiopia and a recent systematic review done in Africa [36, 57, 65]. The possible explanation for this could be exercise reactivate penile tissues and improve erection. And hence, men with low physical activity are at higher risk of developing erectile dysfunction [67].

Another important factor associated with erectile dysfunction in this study is wealth index. Rich diabetic men are 2.62 times at higher risk of developing erectile dysfunction compared to poor ones. This finding is consistent with the research conducted in Ghana [68]. This is due to the fact that being rich puts individuals at higher risk of developing obesity. Being rich can often be seen as an indirect measure of certain characteristics, such as having better access to high-quality healthcare, a wider range of nutritious food options, and the ability to consume energy-dense foods. Additionally, individuals with higher wealth tend to have a lower likelihood of being involved in physically demanding occupations, thus increasing the likelihood of leading a sedentary lifestyle [69–71].

Unlike other studies, this particular study has not shown the association of longer duration of diabetes, type of diabetes, alcohol intake, and cigarette smoking habit with erectile dysfunction. This may be due to socio-cultural conditions of the community.

### Strength and limitations

This study used standard tools like WHO STEPS-wise questionnaires, summary of diabetes self-care activities, international physical activity questionnaire, public health questionnaires and international index of erectile function. Over reporting of the cases might be there hoping for better management. This study didn’t measure micro and macrovascular complications in diabetic patients objectively. Similarly, HbA1C and serum testosterone level were not measured which could have shown long term glycemic control and hormonal level quantitatively. This is because we conducted the research in a poor set-up where there is no access to determine HbA1C and serum testosterone levels for each study participant.

## Conclusion

In conclusion, the prevalence of erectile dysfunction among adult diabetic men is nearly nine out of ten. The risk of erectile dysfunction increased with age, wealth index, and inadequate physical activities. Giventhe higher magnitude of the problem, routine assessment and treatment of erectile dysfunction should be done at the diabetic follow-up clinic regularly. The treating healthcare providers should openly ask and assess the sexual history of adult diabetic men and intervene accordingly. Health education on the importance of adequate physical exercise and adherence to medication should be addressed strictly. Comorbidities should be sought and intervened timely and properly. The Ethiopian ministry of health should give due attention to chronic disease like diabetes mellitus. Separate and updated guideline for diabetic management and its complications should be prepared and distributed to each hospital. Researchers should do more investigations on large sample sizes incorporating laboratory parameters like HgA1c, ankle brachial index and serum testosterone so that updates on risk factors of erectile dysfunction and its management would be incorporated into management guidelines.

### Electronic supplementary material

Below is the link to the electronic supplementary material.

Supplementary Material 1

## Data Availability

The data set analyzed during the current study are available from the corresponding author upon reasonable request.

## List of abbreviations

ACEI: Angiotensin Converting Enzyme Inhibitor
AOR: Adjusted Odds Ratio
ASA: Acetyl Salicylic Acid
BMI: Body Mass Index
BPH: Benign Prostatic Hyperplasia
CCB: Calcium Channel Blocker
CI: Confidence Interval
DM: Diabetes Mellitus
ED: Erectile Dysfunction
GRH: Goba Referral Hospital
HbA1c: A glycated hemoglobin A one c
IIEF: International Index of Erectile Function
MWU: Madda Walabu University
T2DM: Type 2 Diabetes Mellitus
WHO: World Health Organization

## References

[1] Research NI. .o.H.O.o.M.A.o., NIH Consensus Statement. Vol. 10. 1992: National Institutes of Health, Office of Medical Applications of Research.

[2] Peak TC. W.J.G.H., *International Index of Erectile Function* 2017.

[3] Beckman JA, Creager MA (2016). Vascular Complications of Diabetes. Circul Res.

[4] Association AD (2014). Diagnosis and classification of Diabetes Mellitus. Diabetes Care.

[5] Forbes JM, Cooper ME (2013). Mechanisms of diabetic Complications. Physiol Rev.

[6] Brownlee M (2005). The pathobiology of diabetic Complications: a unifying mechanism. Diabetes.

[7] Kasper D, et al. Harrison’s principles of internal medicine, 19e. Volume 1. Mcgraw-hill New York, NY, USA; 2015.

[8] Fasil A, Biadgo B, Abebe M. *Glycemic control and diabetes complications among diabetes mellitus patients attending at University of Gondar Hospital, Northwest Ethiopia* Diabetes, metabolic syndrome and obesity: targets and therapy, 2019. 12: p. 75. 10.2147/DMSO.S185614 PMC6306061 30613158

[9] Jameson J, Kasper FA, Hauser DL, Longo SL, Loscalzo DL. J., *Harrison’s Principles of Internal Medicine, 20e* 2018. ISBN 978-1-259-64404-7; MHID 1-259-64404-9.

[10] Blair M. *Diabetes Mellitus review*. Urol Nurs, 2016. 36(1). 27093761

[11] Rew KT, Heidelbaugh JJ (2016). Erectile dysfunction. Am Family Phys.

[12] Al-Hunayan A, Kehinde A-MM, Thalib EO, Al-Ghorory L. M, *he prevalence and predictors of erectile dysfunction in men with newly diagnosed with type 2 diabetes mellitus* BJU international, 2007. 99(1):130–134. 10.1111/j.1464-410X.2006.06550.x 17026597

[13] Thorve VS (2011). Diabetes-induced erectile dysfunction: epidemiology, pathophysiology and management. J Diabetes Complicat.

[14] Goldstein I (2019). The association of erectile dysfunction with productivity and absenteeism in eight countries globally. Int J Clin Pract.

[15] Burnett AL (2018). Erectile dysfunction: AUA guideline. J Urol.

[16] Jannini EA (2014). Health-related characteristics and unmet needs of men with Erectile Dysfunction: a Survey in five E uropean countries. J Sex Med.

[17] Frost M (2012). Chronic Diseases in elderly men: underreporting and underdiagnosis. Age Ageing.

[18] Goldstein I (2018). Real-world observational results from a database of 48 million men in the United States: relationship of Cardiovascular Disease, Diabetes Mellitus and depression with age and erectile dysfunction. Int J Clin Pract.

[19] Weldesenbet AB, Kebede SA, B.S.J.J.o.I MR, Tusa. *Prevalence of erectile dysfunction and its associated factors among patients with diabetes in Ethiopia: a systematic review and meta-analysis* 2021. 49(2): p. 0300060521993318. 10.1177/0300060521993318 PMC7890740 33583238

[20] Al-Shaiji TF (2022). Breaking the ice of Erectile Dysfunction Taboo: a focus on clinician–patient communication. J Patient Experience.

[21] Goonewardene SS, Pietrzak P, Albala D. *Diagnosis and management of ED*, in *Basic Urological Management*. Springer; 2019. pp. 305–5.

[22] Antonio L (2022). Erectile dysfunction predicts mortality in middle-aged and older men Independent of their sex steroid status. Age Ageing.

[23] Feldman HA (1994). Impotence and its medical and psychosocial correlates: results of the Massachusetts Male Aging Study. J Urol.

[24] Hackett G, et al. The British Society for Sexual Medicine guidelines on male adult testosterone deficiency, with statements for practice. The World Journal of Men’s Health; 2023. p. 41. 10.5534/wjmh.221027 PMC10307648 36876744

[25] O’Donnell AB, Araujo AB, McKinlay JB (2004). The health of normally aging men: the Massachusetts Male Aging Study (1987–2004). Exp Gerontol.

[26] Calzo JP et al. *Erectile dysfunction in a sample of sexually active young adult men from a us cohort: demographic, metabolic and mental health correlates* 2021. 205(2): p. 539–544. 10.1097/JU.0000000000001367 PMC7790854 32935616

[27] Nutalapati S et al. *Association of erectile dysfunction and type II Diabetes Mellitus at a tertiary care centre of south India*. 2020. 14(4): p. 649–53. 10.1016/j.dsx.2020.04.039 32438327

[28] Zeleke M, Hailu D (2021). J.B.e.d. Daka, *Erectile dysfunction and associated factors among diabetic patients at*. Hawassa South Ethiopia.

[29] Yafi FA (2016). Erectile dysfunction. Nat Reviews Disease Primers.

[30] Foroutan SK, Jadid M, Milani (2008). The prevalence of sexual dysfunction among divorce requested. Daneshvar Med.

[31] Saeed R (2021). Prevalence of erectile dysfunction and associated factors among males visiting family medicine clinics in a Tertiary Care Hospital in Karachi. Pakistan.

[32] Mehiret G, Dersie B, J.J.o.D L. *Magnitude and factors contributing to Erectile Dysfunction among Diabetic Men Attending the Diabetic Clinic at Debre Tabor Comprehensive and Specialized, Hospital in North West, Ethiopia 2020, institutional based cross-sectional study*. 2021. 11(3): p. 69–82.

[33] Shiferaw WS, Akalu TY. and Y.A.J.I.j.o.e. Aynalem, *Prevalence of erectile dysfunction in patients with diabetes mellitus and its association with body mass index and Glycated hemoglobin in Africa: a systematic review and meta-analysis* 2020. 2020. 10.1155/2020/5148370 PMC7201640 32411224

[34] KB N et al. *Prevalence and factors associated with erectile dysfunction among adult men in Moshi municipal, Tanzania: community-based study* 2020. 10.1186/s12610-020-00118-0 PMC7709403 33292186

[35] Asaduzzaman M (2020). Frequency and risk factors of Erectile Dysfunction among Bangladeshi adult men with type 2. Diabetes Mellitus.

[36] Asefa A (2019). Prevalence of sexual dysfunction and related factors among Diabetes Mellitus patients. Southwest Ethiopia.

[37] McMahon CG (2019). Current diagnosis and management of erectile dysfunction. Med J Aust.

[38] Beckman N, Ostling WM, Sundh S, Skoog V (2014). Determinants of sexual activity in four birth cohorts of Swedish 70-year-olds examined 1971–2001. J Sex Med.

[39] Hurisa AD, G.Z.J.T.O.P HJ, Negera. Erectile Dysfunct among Diabet Patients Tert Hosp Southwest Ethiopia 2020. 13(1).

[40] Xu Y et al. *Prevalence and correlates of erectile dysfunction in type 2 diabetic men: a population-based cross-sectional study in Chinese men*. 2019. 31(1): p. 9–14. 10.1038/s41443-018-0060-4 30104671

[41] Walle B et al. *Prevalence of erectile dysfunction and associated factors among diabetic men attending the diabetic clinic at Felege Hiwot Referral Hospital, Bahir Dar, North West Ethiopia*, 2016. 2018. 11(1): p. 1–5. 10.1186/s13104-018-3211-2 PMC5815197 29448969

[42] Rosen RC (1997). The international index of erectile function (IIEF): a multidimensional scale for assessment of erectile dysfunction. Urology.

[43] Pelluri R (2021). The role of body mass index or metabolic syndrome components causing depression in women: an observation from weight reduction clinical trial. J Clin Pharm Ther.

[44] Arroll B (2010). Validation of PHQ-2 and PHQ-9 to screen for major depression in the primary care population. The Annals of Family Medicine.

[45] Manea L, Gilbody S, McMillan D (2015). A diagnostic meta-analysis of the Patient Health Questionnaire-9 (PHQ-9) algorithm scoring method as a screen for depression. Gen Hosp Psychiatry.

[46] Lee PH (2011). Validity of the international physical activity questionnaire short form (IPAQ-SF): a systematic review. Int J Behav Nutr Phys Activity.

[47] Jensen MD et al. 2013 *AHA/ACC/TOS guideline for the management of overweight and obesity in adults: a report of the American College of Cardiology/American Heart Association Task Force on Practice Guidelines and The Obesity Society* Journal of the American college of cardiology, 2014. 63(25 Part B): p. 2985–3023. 10.1016/j.jacc.2013.11.004 24239920

[48] Bao X, et al. Proteomic profiles of body Mass Index and Waist-to-hip ratio and their role in incidence of Diabetes. The Journal of Clinical Endocrinology & Metabolism; 2022. 10.1210/clinem/dgac140 PMC9202718 35294966

[49] Seftel AD (2015). J.T.J.o.u., *re: Prevalence of Erectile Dysfunction and Associated Factors among Diabetic Men Attending Diabetic Clinic at Muhimbili National Hospital in Dar-Es-Salaam*. Tanzania.

[50] Mutagaywa RK et al. *Prevalence of erectile dysfunction and associated factors among diabetic men attending diabetic clinic at Muhimbili National Hospital in Dar-Es-Salaam*, Tanzania 2014. 17. 10.11604/pamj.2014.17.227.2695 PMC4145282 25170371

[51] Nisahan B et al. *Erectile dysfunction and associated factors among men with Diabetes Mellitus from a tertiary diabetic center in Northern Sri Lanka*. 2019. 12(1): p. 1–6. 10.1186/s13104-019-4244-x PMC6451292 30953562

[52] Serwaa D (2021). Prevalence and determinant of erectile dysfunction in type II Diabetes Mellitus and healthy men. SciMedicine J.

[53] Tridiantari DK, Saraswati LD, Udiyono AJMJoI (2020). Epidemiology of erectile dysfunction in men with Diabetes Mellitus: a study. Prim Health care Cent Indonesia.

[54] Abeway S et al. *Erectile Dysfunction and Correlates Among Diabetic Men at Dessie Referral Hospital: North Central Ethiopia, 2020* 2020. 13: p. 4201. 10.2147/DMSO.S278384 PMC7654531 33192082

[55] Seid A et al. *Prevalence and determinants of erectile dysfunction among diabetic patients attending in hospitals of central and northwestern zone of Tigray, northern Ethiopia: a cross-sectional study*. 2017. 17(1): p. 1–7. 10.1186/s12902-017-0167-5 PMC5353861 28298205

[56] Degavi G (2021). Determinants and prevalence of Impotence among Diabetic patients in Northwestern hospitals of Nefasit. Eretria.

[57] Shiferaw WS et al. *Risk factors of erectile dysfunction among diabetes patients in Africa: a systematic review and meta-analysis* 2020: p. 100232. 10.1016/j.jcte.2020.100232 PMC7358381 32685380

[58] Corona G et al. *Sexual dysfunction in type 2 Diabetes at diagnosis: progression over time and drug and non-drug correlated factors*. 2016. 11(10): p. e0157915. 10.1371/journal.pone.0157915 PMC5051725 27706160

[59] Kouidrat Y et al. *High prevalence of erectile dysfunction in Diabetes: a systematic review and meta-analysis of 145 studies*. 2017. 34(9): p. 1185–92. 10.1111/dme.13403 28722225

[60] Mahbub M et al. *Frequency and predictors of Erectile Dysfunction in Bangladeshi men with type 2 Diabetes Mellitus: experience from a Tertiary Center*. 2019. 28(1): p. 137–43. 30755562

[61] Tamrakar D (2021). Association between Erectile Dysfunction and Type 2 Diabetes Mellitus.

[62] Pakpahan C et al. *Stem cell therapy and diabetic erectile dysfunction: a critical review*. 2021. 13(10): p. 1549. 10.4252/wjsc.v13.i10.1549 PMC8567456 34786157

[63] Ahsaini M et al. *Prevalence and severity of erectile dysfunction in patients with type 2 diabetes in the Department of Urology at the University Hospital Center Hassan II, Fez, Morocco: a cross-sectional study of 96 cases* 2020. 37: p. 205–205. 10.11604/pamj.2020.37.205.21774 PMC7864255 33598065

[64] Ugwumba FO (2018). Prevalence of, and risk factors for erectile dysfunction in male type 2 diabetic outpatient attendees in Enugu. South East Nigeria.

[65] Shiferawa WS, Akalub TY, Petruckac PM, Habtamu Abera YAA, Arerid. *Risk factors of erectile dysfunction among diabetes patients in Africa: A systematic review and meta-analysis*, in *elsivier*. 2020. p. 9. 10.1016/j.jcte.2020.100232 PMC7358381 32685380

[66] Nguyen CP (2015). Testosterone and age-related hypogonadism—FDA concerns. N Engl J Med.

[67] Duca Y (2019). Erectile dysfunction, physical activity and physical exercise: recommendations for clinical practice. Andrologia.

[68] Owiredu WK (2011). Determinants of sexual dysfunction among clinically diagnosed diabetic patients. Reproductive Biology and Endocrinology.

[69] Tekalegn Y. *Determinants of overweight or obesity among men aged 20–59 years: A case-control study based on the 2016 Ethiopian demographic and health survey* Journal of Obesity, 2021. 2021. 10.1155/2021/6627328 PMC8088365 33981456

[70] Abrha S, Shiferaw S, Ahmed KY (2016). Overweight and obesity and its socio-demographic correlates among urban Ethiopian women: evidence from the 2011 EDHS. BMC Public Health.

[71] Darebo T, Mesfin A, Gebremedhin S (2019). Prevalence and factors associated with overweight and obesity among adults in Hawassa City, southern Ethiopia: a community based cross-sectional study. BMC Obes.

