# Supplementary material for: Magnitude of erectile dysfunction and associated factors among adult diabetic men on follow-up at Goba and Robe hospitals, Bale Zone, South East Ethiopia: hospital-based cross-sectional study
Source: BMC Endocr Disord. 2023 Oct 26;23:236. doi: 10.1186/s12902-023-01489-x (PMC10601257; doi:10.1186/s12902-023-01489-x)
Supplement: Supplementary file 1 — Supplementary Material 1 [file 12902_2023_1489_MOESM1_ESM.docx]

Supplementary Table 1: Alcohol consumption and types of oil used among adult diabetic men attending clinic at Robe and Goba Hospitals

| **Variable** |  | **Frequency** | **percent** |
| --- | --- | --- | --- |
| Current smoking | Yes | 6 | 1.4 |
|  | No | 414 | 98.6 |
| Current daily smoker | Yes | 5 | 1.2 |
|  | No | 1 | 0.2 |
| Ever past smoking | Yes | 64 | 15.2 |
|  | No | 356 | 84.8 |
| Ever alcoholic consumption | Yes | 143 | 34 |
|  | No | 277 | 66 |
| Within the past 12 months alcoholic consumption | Yes | 128 | 30.5 |
|  | No | 15 | 3.5 |
| Frequency of having at least one alcoholic drink during the past 12 months | 5-6 days per week | 7 | 1.7 |
|  | 1-4 days per week | 10 | 2.4 |
|  | 1-3 days per month | 74 | 17.6 |
|  | Less than once a month | 37 | 8.8 |
| Alcoholic consumption within the past 30 days | Yes | 95 | 22.6 |
|  | No | 48 | 11.4 |
| Types of oil | Vegetable oil | 373 | 88.8 |
|  | Lard or suet | 39 | 9.3 |
|  | Butter or ghee | 5 | 1.2 |
|  | Don’t know | 3 | 0.7 |
| Meals eaten that were not prepared at home per week | not eat outside | 229 | 54.5 |
|  | Eats ≥1 outside | 191 | 45.5 |
| Skip breakfast per week | No skip | 240 | 57.1 |
|  | Skip ≥1 per week | 180 | 42.9 |
